# Self-supervised artificial intelligence predicts recurrence, metastasis and disease specific death from primary cutaneous squamous cell carcinoma at diagnosis

**DOI:** 10.21203/rs.3.rs-3607399/v1

**Published:** 2023-12-13

**Authors:** Nicolas Coudray, Michelle C. Juarez, Maressa C. Criscito, Adalberto Claudio Quiros, Reason Wilken, Stephanie R. Jackson Cullison, Mary L. Stevenson, Nicole A. Doudican, Ke Yuan, Jamie D. Aquino, Daniel M. Klufas, Jeffrey P. North, Siegrid S. Yu, Fadi Murad, Emily Ruiz, Chrysalyne D. Schmults, Aristotelis Tsirigos, John A. Carucci

**Affiliations:** 1 Applied Bioinformatics Laboratories, New York University School of Medicine, New York, NY, USA; 2 Department of Cell Biology, New York University School of Medicine, New York, NY, USA; 3 The Ronald O. Perelman Department of Dermatology, New York University Grossman School of Medicine, New York, NY, USA; 4 School of Computing Science, University of Glasgow, Glasgow, Scotland, UK; 5 Department of Dermatology, Northwell Health, New York, NY, USA; 6 Department of Dermatology, Thomas Jefferson University, Philadelphia, PA, USA; 7 School of Computing Science, University of Glasgow, Glasgow, Scotland, UK (Ke Yuan); 8 School of Cancer Sciences, University of Glasgow, Glasgow, Scotland, UK (Ke Yuan); 9 Cancer Research UK Beatson Institute, Glasgow, Scotland, UK (Ke Yuan); 10 Department of Dermatology, University of California, San Francisco, San Francisco, CA, USA; 11 Department of Dermatology, Brigham and Women’s Hospital, Harvard Medical School, Boston, MA, USA; 12 Department of Pathology, New York University School of Medicine, New York, NY, USA

## Abstract

Primary cutaneous squamous cell carcinoma (cSCC) is responsible for ~10,000 deaths annually in the United States. Stratification of risk of poor outcome (PO) including recurrence, metastasis and disease specific death (DSD) at initial biopsy would significantly impact clinical decision-making during the initial post operative period where intervention has been shown to be most effective. In this multi-institutional study, we developed a state-of-the-art self-supervised deep-learning approach with interpretability power and demonstrated its ability to predict poor outcomes of cSCCs at the time of initial biopsy. By highlighting histomorphological phenotypes, our approach demonstrates that poor differentiation and deep invasion correlate with poor prognosis. Our approach is particularly efficient at defining poor outcome risk in Brigham and Women’s Hospital (BWH) T2a and American Joint Committee on Cancer (AJCC) T2 cSCCs. This bridges a significant gap in our ability to assess risk among T2a/T2 cSCCs and may be useful in defining patients at highest risk of poor outcome at the time of diagnosis. Early identification of highest-risk patients could signal implementation of more stringent surveillance, rigorous diagnostic work up and identify patients who might best respond to early postoperative adjunctive treatment.

## Introduction

Cutaneous squamous cell carcinoma (cSCC) is the second most common human cancer, with an estimated incidence of over 1 million cases in the United States and an increasing worldwide incidence over the past 20 years(1–4). While most cSCCs portend a good prognosis, a subset of tumors are associated with poor outcomes (PO)(5, 6) including local recurrence (LR), nodal metastasis (NM), distant metastasis (DM), and disease-specific death (DSD). cSCC causes the majority of keratinocyte carcinoma (KC) deaths in the United States (US); it is estimated that ~10,000 patients die annually from cSCC, which is similar to DSD rates from other cancers such as leukemia, non-hodgkin lymphoma, and melanoma(7). Respectively, nodal metastasis and cSCC-specific death occur in approximately 5% and 2% of patients(8, 9). While PO are rare, these values are likely underestimations given that there is currently no national cancer registry data for cSCCs that accurately assess incidence and prevalence. In addition, we must recognize that relatively small percentages of poor outcomes from extraordinarily large numbers of patients with cSCC result in significant numbers of patients with metastases and disease specific death. This in turns results in significant morbidity, mortality and public health cost burden that may be avoidable if highest risk patients are identified early in their course and receive appropriate adjuvant intervention (10) Furthermore, many epidemiologic studies(11, 12) often combine data on keratinocytic carcinomas (basal cell and squamous cell carcinomas) and thus incidence and prevalence rates are widely variable. With an increasing incidence of cSCC, PO may also be rising, making this an underrecognized, although significant, public health entity.

Identifying patients at risk for poor outcome at time of diagnosis can be challenging. Commonly used cSCC staging systems include the American Joint Committee on Cancer Staging Manual 8th edition (AJCC 8)(13, 14) and the Brigham and Women’s Hospital (BWH) Staging System. The AJCC 8 system utilizes the following factors for cSCC tumor (T) staging: tumor size, deep invasion, perineural invasion (PNI) or evidence of bone invasion. The BWH staging model includes tumor size, poor differentiation, PNI, extension beyond subcutaneous fat and assigns a T stage based on the number of high risk features present. While most POs occur in high stage tumors (BWH T2b and AJCC 8 T3 and above), 25% of POs still occur in low stage tumors, particularly T2a/T2, highlighting the heterogeneity in outcomes, and concomitant difficulties with risk stratification, in this particular subset(15). Thus, prediction of patients at risk for poor outcome, even in lower stage tumors, is imperative as it may affect management in terms of work up, treatment,postoperative surveillance and early implementation of adjuvant therapy(16, 17). Importantly, identification of which patients may be at risk for POs in this low stage subgroup is ill-defined in the literature and remains under urgent investigation.

While gene expression has predictive power(18, 19), such technology may not be accessible to all patients and is associated with high cost. Visual inspection of the distinct histopathological features found in cSCC, on the other hand, remains important in predicting risk of progression and its role in predicting risk of PO in low staged tumors requires further elucidation(20). Microscopic analysis using hematoxylin and eosin (H&E) stained tissue is the gold standard for diagnosing cSCC and is useful in identifying high risk features such as depth of invasion, PNI, and degree of differentiation. However, light microscopy has a limited role in determining prognosis itself, given potential for sampling error as well as inherent inter-reader variability in histopathologic features. Recently, supervised machine learning algorithms have been used in the cutaneous melanoma realm to identify prognostically important features, response to immunotherapy, one-year disease-free survival and mutation prediction with promising results(21–24). Current studies looking at machine learning for KC, such as squamous cell carcinoma are, however, limited. Few studies have investigated the use of artificial intelligence (AI) on whole slide images (WSI) of cSCC: Recently, *Knuutila et al*.(25) trained a supervised ResNet architecture to show that artificial intelligence can predict the risk of metastasis from primary tumor slides of cSCC, but this approach was not amenable to describing which features were used by the classifier.

**Figure 1.**
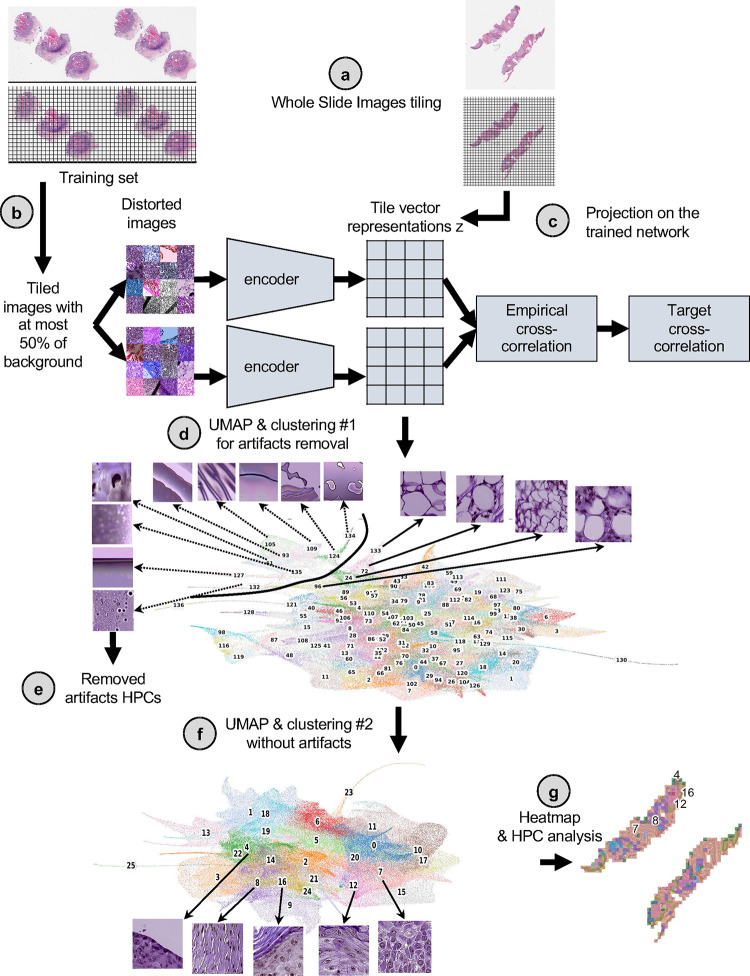
Adaptation of the self-supervised Histological Phenotype Learning pipeline to study cutaneous squamous cell cancer. **a.** The slides were first tiled into smaller images of 224 × 224 pixels at 0.5 um/pixel (equivalent to a magnification of 20x). **b.** A subset of those tiles were used to train the self-supervised Barlow-Twins architecture. **c.** Once trained, all the tiles from the three cohorts were then projected onto the trained network to extract their tile vector representations z, a 128 vector coding each image. **d.** Those vector representations are then over-clustered using the Leiden approach in order to get homogeneous clusters (called Histomorphological Phenotype Clusters, HPC) and visually identify artifacts from tissue representations. In this UMAP of the tile vector representation z, each dot represents a tile, and each color a different HPC. **e.** Tiles belonging to HPCs identified as highly enriched in artifacts are removed from the study. **f.** The cleaned dataset is then subject to more detailed analysis and subjected to a new round of Leiden clustering. This UMAP of the cleaned tile vector representations z shows 26 HPCs corresponding to 26 groups of self-identified phenotypes, and representative tile for the top 5 clusters corresponding to the example slides in panel **c**. **g.** The resulting HPCs can then be used to generate heatmaps showing simplified slide representations and analyzed to identify potential correlations between those phenotypes identified by the self-supervised approach and patients’ outcome. Here, the heat maps corresponding to the example slide section in panel **a** is shown, with the top 5 clusters numbered and corresponding to the ones in panel **f**. All tiles are shown after Reinhard’s color normalization(48).

Herein, our goal was to investigate the histomorphological features associated with cSCC outcomes via a self-supervised deep-learning approach using images from WSI collected from shave or punch biopsy specimens of primary cSCC tumors. While supervised approaches are trained to directly learn specific labels (which can often be time-consuming to obtain, require expertise, and may generate bias)(26–28), self-supervised approaches achieve self-discovery of common patterns across unlabeled datasets (29–32). Supervised approaches are also often described as black boxes whose decision process is difficult to interpret(33, 34), which despite an effort to develop interpretation strategies(35), is hindering its acceptance by humans and regulatory approval organizations(34). Investigating an unbiased and interpretable strategy for prediction of outcome is therefore crucial, and self-supervised learning paradigms currently offer great opportunities for the development of AI in research and medicine(36). In this study, we included 163 patients from three academic institutions (Supplementary Figure 1a) and based the core of our study on a Histological Phenotype Pipeline (HPL)(29). This pipeline (Figure 1) has recently been developed in a lung cancer study and has shown to cluster significant WSI features in a self-supervised manner, leading to promising results in terms of subtype classification and survival prediction(29). Furthermore, HPL provides an additional layer of interpretability. Supervised approaches were also considered either as a baseline comparison or as a means to extract further information from the images (Supplementary Figure 1b).

## Results

### Self-supervised learning highlights histomorphological phenotypes associated with poor and good outcomes

Slides were collected from three institutions (Supplementary Table 1, Supplementary Figure 1a): New York University (NYU), University of California San Francisco (UCSF) and Brigham and Women’s Hospital (BWH), along with clinical information regarding whether the patient developed good or poor outcome. Fourteen slides were excluded due to missing data or poor slide quality (Supplementary Figure 5). In total, slides from 163 patients diagnosed with cSCC on initial biopsy who developed good or poor outcomes were included (NYU: 38 patients, n=31 and 7, respectively; UCSF: 85 patients, n=58 and 27, respectively; BWH: 40 patients, n=20 and 20, respectively).

Clinical outcome data was notated as a binary label of good vs. poor outcome, with good outcome indicating no evidence of disease at most recent follow-up and PO representing local recurrence, nodal metastases, distant metastases or disease-specific death. Furthermore, disease-free survival (DFS) data was available for the NYU and UCSF datasets (median follow-up, 38.0 and 32.7 months), allowing us to perform Cox regression models with these cohorts. Further patient information regarding age, tumor stage, size, was not available for the UCSF and BWH cohorts. To objectively identify phenotypes which could potentially be predictive of PO, we used a pipeline based on the Barlow-Twins self-supervised approach (37) as described in Figure 1 (Method for details). This pipeline has been shown to successfully identify clusters of meaningful phenotypes on lung adenocarcinoma (such as different histological subtypes and types of tissues), and link them to overall and recurrence-free survival(29). The method works on 224×224 pixel tiles tessellated from WSIs (Figure 1a). After having trained a self-supervised Barlow-Twins(29, 37) based model (Figure 1b, Supplementary Figure 2a), the tiles of the entire dataset are projected onto the latent space of the trained network to extract tile vector representations z, z being a 128 dimension vector coding the information contained in a tile (Figure 1c). The Barlow-Twins model runs into two identical encoders, batches of samples originating from similar tiles but distorted in different ways. In trying to minimize the empirical cross-correlation between the embeddings on those two networks and the target cross-correlation, images projected into that network will eventually lead to tile vector representations that are more similar to each other if the corresponding images carry similar features. To simplify the analysis of the vector representations, tiles sharing similar phenotypes are clustered into groups called HPC (Histomorphological Phenotype Clusters) using the Leiden algorithm, which has the advantage of being a community detection algorithm with hierarchical clustering and can identify groups of nodes as well as connections between the entities.

Tile images sharing similar properties are therefore expected to share close *z* tile vector representations and can be represented via a UMAP dimension reduction plot (Figure 1d). Applying the Leiden clustering algorithm(38) on the tile vector representations and selecting a random 100 tiles from each cluster constitutes an efficient way to quickly assign labels to a wide range of tiles and slides.

To remove the tiles showing artifacts (such as air bubbles, blurring, dust), we applied a first Leiden clustering step with a high enough resolution (r=7) to increase the chance of clusters to be homogenous (higher resolutions leads to more clusters and increases the possibility of over-clustering). We identified 9 clusters containing artifacts which were removed from the dataset. Those artifacts are all contained within regions protruding from the rest of the UMAP (Figure 1e). Next, another round of Leiden clustering was applied to the remaining tiles (Figure 1f). Finally, each HPC can be mapped back to the slide of each patient (Figure 1g). Each patient can be described by a patient vector representation which is embedded in the percentage of tiles associated with each HPC.

**Figure 2.**
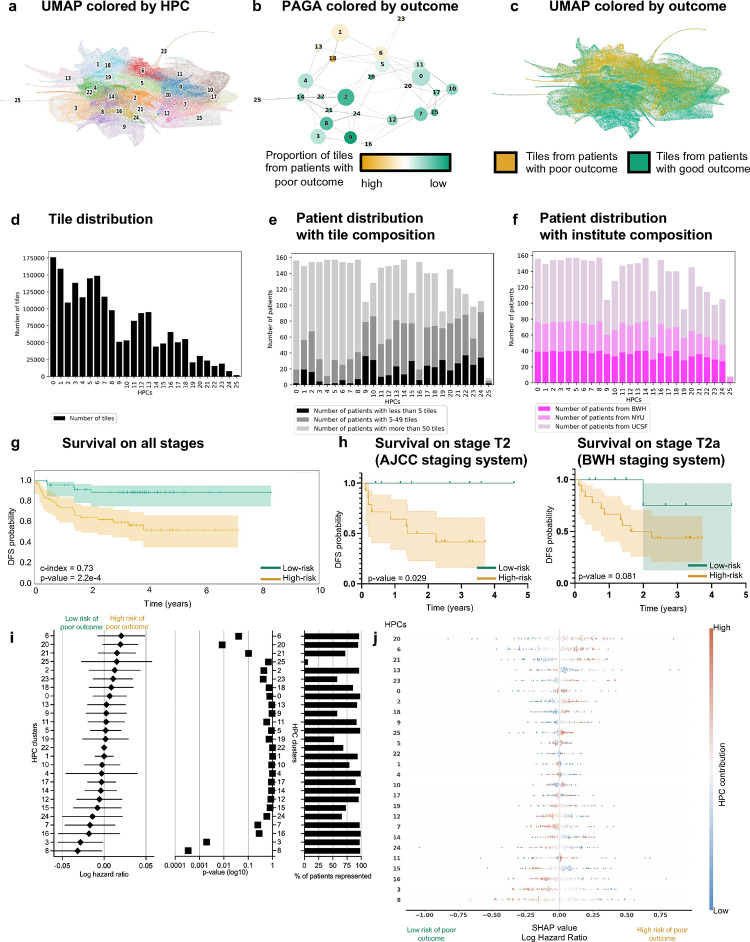
Self-supervised approach generates clusters enriched in tiles from patients with poor outcome, with good representation of the three cohorts, and achieving a c-index of 0.73 in disease-free survival prediction while providing tile clusters important for that prediction. **a.** UMAP with the 26 Leiden clusters found at resolution 0.75. **b.** PAGA representation of the Leiden clusters with node connections. The size of the nodes is proportional to the number of tiles and their color is proportional to the proportion of tiles associated with good/poor outcome patients. **c.** UMAP with colors showing tiles associated with good/poor outcome patients (green/orange). Each dot is a tile. **d.** Tile distributions on the HPCs. **e.** Number of patients present in the different HPCs, stratified by the number of tiles for each patient. **f.** Number of patients present in the different HPCs, stratified by the enrolling institution (maximum number of patients of 163). **g.** Kaplan-Meier curve of predicted high and low risk patients of having poor outcome from the self-supervised HPL approach using a Cox regression. **h.** Kaplan-Meier curves of predicted high- and low-risk patients of having poor outcome from the self-supervised HPL approach using a Cox regression, showing the stages from subsets of panel 2g (error bars correspond to 95% confidence interval)**. i.** Forest plot with log hazard ratio of Cox proportional hazards model over the three-fold cross-validation. For each HPC, the log hazard ratio, p-value and percentage of patients with at least one tile belonging to that HPC are shown from left to right. Coefficients were averaged across folds and p-values combined with Fisher’s probability test. **j.** Interpretability of the HPCs via SHAP shows, at the top, which HPCs favor higher risk of poor outcome prediction when enrichment in tiles is present for patients, and which favor prediction for good outcome (bottom).

Following the strategy explained in the method section to reduce overfitting, we froze a Leiden cluster configuration corresponding to a resolution r=0.75, leading to splitting the dataset into 26 HPCs (Figure 2a). A PAGA (PArtition-based Graph Abstraction) representation (Figure 2b) illustrates how the clusters at the top of the graph and of the corresponding UMAP (Figure 2c) seem more enriched in tiles associated with risk of poor outcome. As expected, at the selected resolution, phenotypes are present in different proportions and the relative size of HPCs vary considerably (Figure 2d). However, we can see that patients are well represented, and many of those phenotypes are present to various degrees in many of the 163 patients (Figure 2e), with the exception of HPC 25. Similar observation is done regarding the representativity of the different institutions (Figure 2f, Supplementary Figure 2b), showing thereby no overfitting.

Using the HPL pipeline, each patient can be described by the percentage of tiles contained in each HPC, which in turn can be used as an input into regression models to estimate the HPC outcome prediction power. To assess the variability and impact of Leiden clustering on prediction of outcome, we also ran three-fold cross-validation log regression for binary classification of poor versus good outcome (Supplementary Figure 2d) and a Cox regressions for survival prediction analysis (Supplementary Figure 2e) at various resolutions. We observe that the resolution of r=0.75 selected above also allows for successful predictions in both approaches while preventing overfitting.

### Self-supervised learning leads to c-index of 0.73 for DFS prediction and AUC~0.7 for binary classification

As mentioned, each patient can be described by the proportion of tiles assigned to each HPC, and this simplified phenotype description can in turn be used to study outcome predictability. Using a log regression approach, we investigated the potential to predict the binary outcome from those HPCs. The good versus poor outcome binary classification run on the 3 datasets available resulted in an average 3-fold cross-validation AUROC of 0.724 (validation set) and 0.689 (test set) (Supplementary Figure 3a). This result, as based on a self-supervised approach, allows for an unbiased selection of clusters of tiles which are identified as the most relevant to achieve the classification. It also provides an easy way to identify phenotypes important for such prediction, such as a forest plot analysis which shows the contribution of the main clusters to this prediction (Supplementary Figure 3b).

As a comparison, we trained the supervised network inception v3, which relies on selected labeled regions. Manual free-text annotations based on consensus agreement were performed by three board certified Mohs micrographic surgeons (M.C., S.R.J, R.W.) and a senior dermatology resident (M.J.) using Aperio’s Imagescope interface. Consensus was achieved if all reviewers agreed with the annotation features. We followed the pipeline similarly used by Johannet *et al.* (22) in their study of melanoma. Here, we first trained the algorithm (Supplementary Figure 3c, Supplementary Table 2–3) to identify the regions manually annotated (normal skin, squamous cell carcinoma *in situ*, invasive squamous cell carcinoma, other, and artifact). Annotations in the ‘other’ group included dermis, fat, glandular tissue, smooth muscle, cartilage, inflammatory infiltrates, and the presence of ortho and hyperkeratosis. The artifact annotation included negative white space, bubbles or pen markings, as mentioned above. Second, we checked whether a selected region (invasive cSCC) or a set of regions of interest (normal, *in situ*, invasive) could be used to predict a binary outcome (Supplementary Figure 3d). Such a supervised approach achieved performances of AUROC=0.675 on invasive cSCC and 0.671 on the set of regions of interest (normal, *in situ* and invasive combined). In addition to performing slightly worse than the self-supervised approach (Supplementary Figure 4a and more metrics in Supplementary Table 4–5), this two-step method relies on manual annotations, choices of regions from which the prediction should be made, and little possibility to directly interpret how the model made its decision or what subsets of phenotypes were used. These three bottlenecks are all addressed by the self-supervised approach as described in the next section.

More interestingly, with the two datasets where the DFS data are available, we obtain a DFS prediction with a Harrell’s c-index of 0.73 (0.72 Uno’s c-index), and p-value of 2.2e-4 (Figure 2g). A forest plot (Figure 2i) and SHAP plot (Figure 2j) were computed to interpret the contributions of the different HPCs to this prediction and understand which phenotypes influence the model’s prediction. This approach is particularly effective at differentiating poor from good outcome in a subset of BWH T2a and AJCC-8 T2 tumors respectively, which currently face significant outcome heterogeneity making it difficult to predict outcome (Figure 2h). Note that the Kaplan-Meier plots extrapolated from the poor outcome probabilities generated by supervised classifiers show much lower performances (Supplementary Figure 4b–c). This self-supervised approach could potentially address the gap in the current staging systems caused by the relative lack of outcome homogeneity within AJCC T2 and BWH T2a groups.

### Cluster interpretation highlights histomorphological phenotypes linked with increased risk of local recurrence, metastasis and good outcome

**Figure 3.**
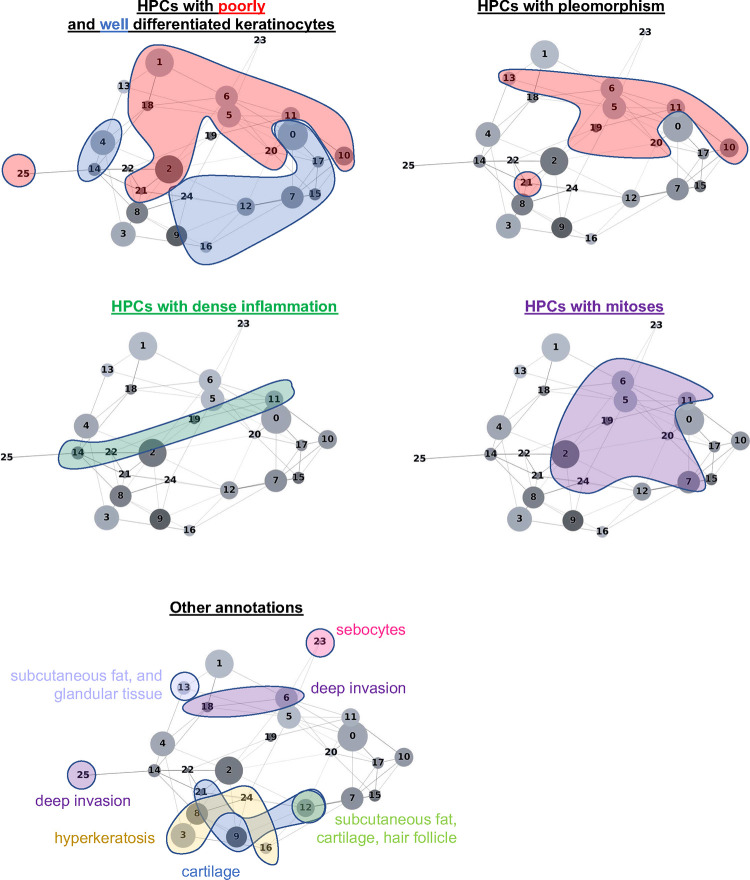
PAGA graph shows a coherent organization of features found on cutaneous squamous cell carcinoma whole slide images. Annotations provided by a group of Mohs surgeons, of which included 100 tiles randomly selected for each HPC (annotation taken from Supplementary Table 6) and are projected on the PAGA graph from Figure 2C.

The HPL self-supervised approach provides interpretability power that allows us to identify how HPCs are weighted in terms of lower or higher risk of overall PO in our Cox regression model (DFS), or in terms of good/poor outcome in the log regression model. First, 100 random tiles were selected from each HPC and visually analyzed by three board certified Mohs micrographic surgeons (M.C., S.R.J, R.W.) and a senior dermatology resident (M.J.). The details of these observations are shown in Supplementary Table 6 (see Supplementary Figure 6 for a subset of randomly selected tiles). When we project those observations on the Partition-based Graph Abstraction (PAGA Figure 3), we observe that HPCs sharing similar phenotypes are linked and located in specific and coherent regions, confirming the coherence of the HPL representation.

**Figure 4.**
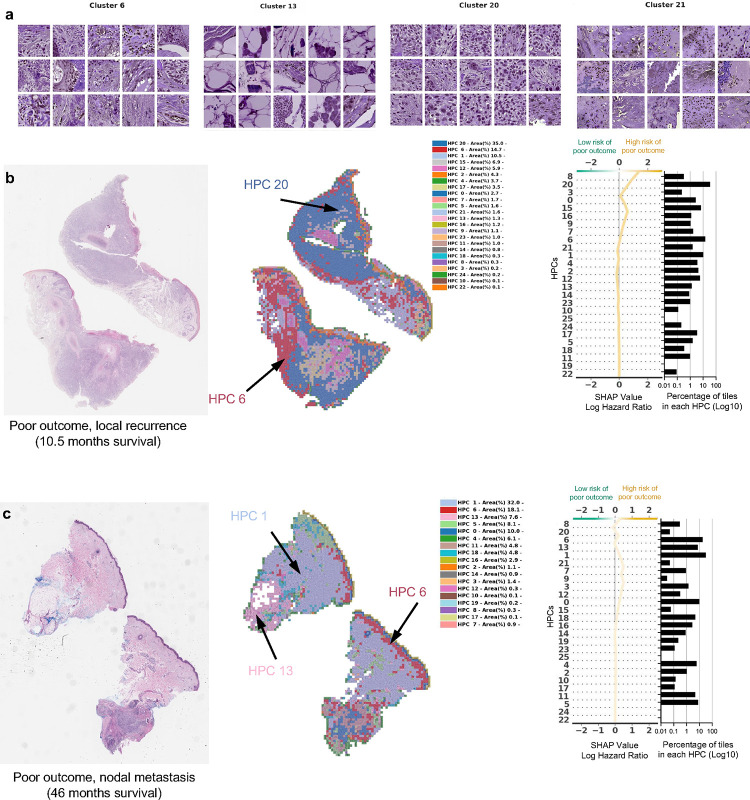
Example of tiles from HPCs associated with higher risk of poor outcome. **a.** Example of tiles randomly selected from certain HPCs leading to risk prediction of poor outcome. **b-c.** Examples of data from patients with poor outcome shortly after surgery (10.5 months, local recurrence) and with poor outcome a few years after surgery (46 months, nodal metastasis). For each case, a small portion of the original slide is shown as well as the corresponding heatmap and the associated SHAP decision plot. The color of the heatmap shows the HPC associated with each tile, with the proportion of tile belonging to each HPC shown in the legend (percentages computed over the whole slide(s) available for each patient). The top of the SHAP decision plot shows the predicted value which determines the color of the curve. Reading from bottom to top, the SHAP values for each HPC are cumulatively summed, and the HPCs are ordered according to the absolute SHAP weight. On the right, the proportion of tiles associated with each cluster is shown on a Log10 scale. All tiles are shown after Reinhard’s color normalization (48).

**Figure 5.**
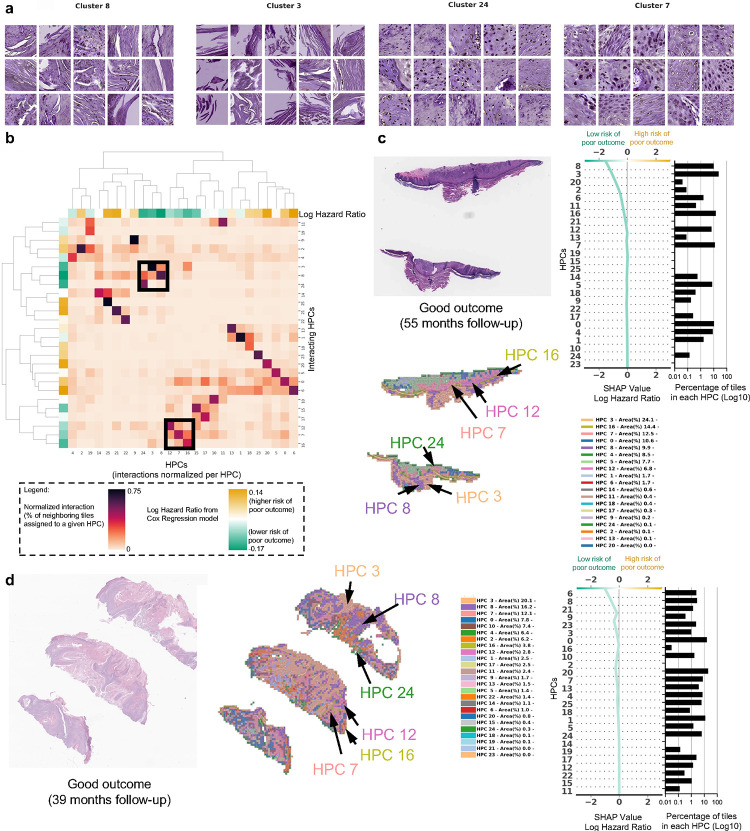
Example of tiles from HPCs associated with lower risk of poor outcome. **a.** Example of tiles randomly selected from certain HPCs leading to prediction of good outcome. **b.** The interaction analysis between HPCs shows two groups of HPCs which tend to be adjacent on slides; each column shows the normalized proportion of interactions each tile associated with a given HPC has with HPCs associated with its adjacent tiles. The dendrograms correspond to bi-hierarchical clustering of HPCs. **c-d.** Examples of data from patients who have not recurred and have been followed for more than three years. For each case, a small portion of the original slide is shown as well as the corresponding heatmap and the associated SHAP decision plot. The color of the heatmap shows the HPC associated with each tile, with the proportion of tile belonging to each HPC shown in the legend (percentages computed over the whole slide(s) available for each patient). The top of the SHAP decision plot shows the predicted value which determines the color of the curve. Reading from bottom to top, the SHAP values for each HPC are cumulatively summed, and the HPCs are ordered according to the absolute SHAP weight. On the right, the proportion of tiles associated with each cluster is shown on a Log10 scale. All tiles are shown after Reinhard’s color normalization (48).

In Figure 4 and in Figure 5, we show examples of HPCs and tiles associated with higher and lower risk of PO by our Cox model from Figure 2g.

In Figure 4a we show randomly selected tiles from the HPCs having the highest influence on the c-index for prediction of higher risk of PO, and in Figure 4b–c, three heatmaps of patients with PO show the HPCs composition projected on sections of the WSI. For the patient in Figure 4b, for example, the tumor recurred after 10.5 months and its associated WSI demonstrated high enrichments in HPCs 1, 6 and 20, the latter two with a high SHAP log hazard ratio value synonymous with higher risk of PO. The HPC 6 shows deep invasion of poorly differentiated keratinocytes with mitoses. HPC 20 shows poorly differentiated and pleomorphic keratinocytes with mitoses, and HPC 1 similarly demonstrates poorly differentiated keratinocytes, which have been shown in prior studies to correlate with POs, such as local recurrence(39).

The WSI in Figure 4c shows a high presence of HPCs 0, 1, 5, 6 and 13, the latter 2 showing a high SHAP log hazard ratio value. HPC 13 shows some pleomorphic features as well as deeply infiltrative tumor cells, of which is a feature associated with POs. Similarly HPC 6 demonstrates deep invasion, poor differentiation, and significant atypia, factors that are recognized to contribute to POs(39). The SHAP decision plots resulting from our study show how the enrichment or depletion of tiles from certain HPCs weigh into the final decision for a patient whose tumor recurred shortly after surgery (LR, Figure 4b) and for a patient recurring 46 months after surgery (NM, Figure 4c).

In Figure 5a we show randomly selected tiles for the HPCs having the highest influence on the c-index for prediction of a lower-risk of PO. In Figure 5b, an analysis shows clusters of HPCs which tend to be adjacent on a slide, by checking, for each tile belonging to a given HPCs, what HPCs were associated with its adjacent tiles. Two groups of HPCs identified as lower risk of PO were seen as having relatively high interactions: HPCs 7, 12 and 16 as one group and HPCs 3, 8 and 24 as the other. A commonality of all these HPCS was the presence of well-differentiated keratinocytes and the lack of atypia or pleomorphism, which would be expected for a tumor with good outcome. In Figure 5c–d, we show the slides associated with two patients who were followed up for more than three years with no evidence of disease. Those slides show the presence of HPCs 3,8 and 24 seen in Figure 5b, as well as HPCs 7, 12 and 16. Similarly, it is likely that the good differentiation, lack of pleomorphism and relatively non-specific features of hyperkeratosis can be attributed to these findings.

**Figure 6.**
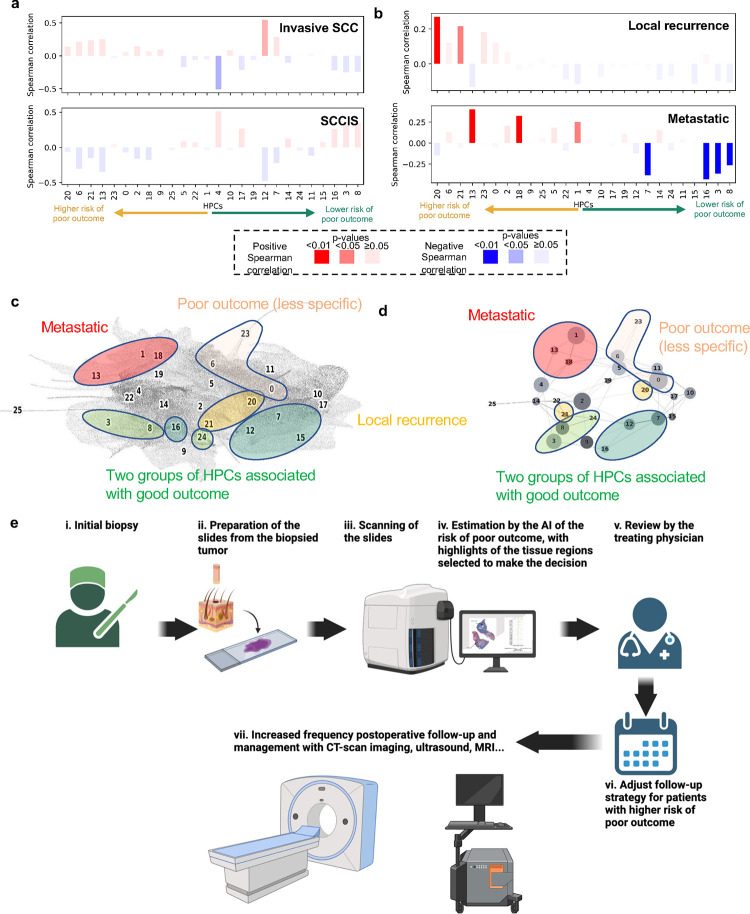
Specific HPCs are correlated with pathologic diagnosis or type of poor outcome. **a.** Spearman correlation between the HPCs and the whole slide pathologic diagnosis available for the NYU slides. **b.** Spearman correlation between the HPCs and the type of poor outcome (LR for local recurrence versus overall metastatic). The box plots show the Spearman correlation (positive in red, negative in blue) with the intensity modulated by the p-value. For simplicity, x-axis is ordered as the y-axis of the SHAP plot in Figure 2c. **c-d.** Projection on the UMAP and PAGA graph of the HPCs associated with high and low risk of poor outcome. HPCs associated with higher risk of metastasis (nodal metastasis (NM) and distant metastasis (DM)) are shown in red, those associated with local recurrence in dark yellow (taken from panel **b**), and those associated with non-specific poor outcome in paler yellow. The two clusters of likely correlated HPCs associated with good outcome are shown in two shades of green (from Figure 5b). **e**. Ultimately, we anticipate such a deep-learning tool, which identifies patients at higher risk with poor outcome and provides histomorphological interpretability, could assist treating physicians in making decisions on an increased post-operative follow-up and management strategy.

For the NYU dataset, whole-slide pathologic diagnosis information was available. We investigated whether HPCs were enriched in patients showing certain diagnoses. Although a slight trend revealed that slides from patients diagnosed with invasive cSCC were likely more enriched in HPCs associated with higher risk of PO, those diagnosed with *in situ* disease were more correlated with HPCs associated with better outcome. However, these results did not reach statistical significance (Figure 6a). A similar analysis was performed to determine if the HPCs could be used to predict the type of PO (Figure 6b). Interestingly, HPCs 20, 21 and to a lesser degree 23 seem to be predictive of local recurrence, whereas HPCs 13, 18 and 1 may be predictive of overall metastasis. Overall, all these HPCs share a common finding of poor differentiation, significant pleomorphism and atypia, and deep invasion.

## Discussion

In this study, we demonstrate that the self-supervised approach of HPL can be successfully applied to analyze sets of cSCC WSIs from initial biopsy samples from different institutions, grouping in a coherent manner a variety of histopathological features linked to good and POs. The ability to obtain prognostic information from biopsy slides alone is significant as it may guide clinical decision making regarding treatment and surveillance of patients with potential PO. The HPCs identified were used to predict the good versus poor outcome with an area under the curve (AUC) of ~0.7 and the disease-free survival (DFS) with a c-index of 0.73 (p-value= 2.2e-4). The performance remains compelling in a subset of AJCC-8 T2 and BWH T2a tumors (p=0.029 and 0.081, respectively). The performance achieved here can also be placed in the context of other methods to which it could be potentially combined to further refine the precision of the outcome. For example, Zhao *et al.* (19) showed that protein expression of AXIN2 and SNAIL have a c-index of 0.69 in predicting recurrence-free survival, and that, although their available clinicopathological data alone had little prediction power (c-index of 0.40), the c-index was increased to 0.75 when combining clinicopathological with the protein expression. Using supervised deep-learning architectures, few studies have explored the predictability of cSCC from WSIs, all of which were unable to pinpoint which features were used by the algorithm to make the decision. Focusing on prediction of metastasis in a cohort of 104 patients harboring cSCC, *Knuutila et al.* (25) achieved AUCs within 0.629–0.689, performing better (AUC=0.747) when restricting the study to those that recurred rapidly (within 180 days). This study was done using either the whole slide or tumor regions manually annotated by pathologists. On the other hand, using two cohorts of 54 melanoma patients, *Comes et al.* (24) achieved AUCs=0.667–0.695 in predicting the one-year disease free survival using regions of interest manually pre-selected by pathologists. In addition to its performance, the advantages of the HPL pipeline, initially developed on lung cancer(29), is that its training is self-supervised and does not require any manual pre-annotations. Furthermore, it provides an additional layer of interpretation highlighting which phenotypes weighed in favor of higher or lower risk prediction.

In our study we found that enrichment in HPCs 3 and 8 correlated with a prediction of good outcome from cSCC. Both HPCs demonstrate well-differentiated keratinocytes, lack of atypia or pleomorphism, and the relatively non-specific presence of hyperkeratosis. Alternatively, enrichment in HPCs 7, 12 and 16, which feature well-differentiated cells and lacked pleomorphism, deep invasion, or significant atypia, also correlated towards a lower risk of PO. Additionally, among the HPCs identified that correlated with higher risk of PO, the two major phenotypes identified were severe pleomorphism with poor differentiation and deep invasion. This result is consistent not only with previous studies but also with the current BWH and AJCC-8 staging systems(39). In a recent meta analysis, *Zakhem et al.* revealed that tumors with invasion beyond the subcutaneous fat were associated with a statistically significant risk of LR and DSD(40–42). Moreover, ulcerated tumors, poorly differentiated tumors, PNI, lymphovascular invasion, desmoplastic stroma and immunosuppression are all significantly associated with POs (39, 43).These attributes have been defined as key defining features of aggressive behavior by cSCC and some are considered in staging. It is for this reason that the ability of our machine learning algorithm to detect these features, at time of biopsy, is significant. Our system may offer a standardized method for feature identification given the potential for inherent inter-reader variability in identification of high risk histopathologic features by dermatopathologists. Poor differentiation and invasion beyond the subcutaneous fat have been associated with an increased risk of metastasis(44). In our study, it is interesting to note that HPL identified phenotype enrichments specific to the type of PO of the patients whose tumor recurred (local recurrence or overall metastatic) in WSI from initial biopsy specimens. For example, HPCs 20, 21 and 23 were predictive of local recurrence, while HPCs 13, 18 and 1 were predictive of overall metastasis. While a commonality of all these HPCs was significant pleomorphism, atypia, and poor differentiation, those predictive of metastasis were also associated with deeper invasion. Again, this finding is in line with current staging systems that denote a tumor as higher risk with an increasing number of high risk features such as poor differentiation, deep invasion, or PNI. More interestingly, the different clusters identified in this study and their association with certain types of outcomes are located in well-defined and coherently connected regions of the UMAP and of the PAGA graphs (Figure 6c–d). The two sets of HPCs associated with good outcome (HPCs 3, 8 and 24 as one group, and 7, 12 and 16 on the other) and identified as having high numbers of interactions on the slides are each connected in the PAGA and located on the lower right side of the UMAP. On the other hand, the top-right side of the UMAP is dominated by HPCs associated with POs. Those associated with higher risk of overall metastasis (HPCs 1, 13 and 18) were all connected in the PAGA, therefore confirming some connections between the associated phenotypes.

In this study, we uniquely used a self-supervised learning followed by community-based clustering to achieve a c-index of 0.73 (p-value= 2.2e-4) in predicting cSCC-free survival, while describing the phenotypes of the clusters weighing the most in these findings. Predictive clusters for PO included those with poor differentiation whereas lack thereof, enrichment in non-specific hyperkeratosis without atypia tended to favor prediction of good prognosis. Importantly, we demonstrate significant potential to optimize clinical decision-making in that this approach is particularly efficient at differentiating PO risk in low stage tumors (BWH T2a and AJCC-8 T2 tumors). This addresses a large gap in the literature relating to outcome homogeneity between low stage tumors (BWH T2a and AJCC T2) given that ~25% of POs occur in low stage tumors(15). Identification of highest risk patients would allow for rationally based, focused clinical follow up and eventually may lead to the development of algorithms for further imaging and work up.

Overall, these results suggest that prognostication of cSSC can benefit from self-supervised learning to not only assist clinicians in predicting outcomes but also highlight histomorphological patterns associated with these outcomes. These findings play a significant role in patient care, as prognostic information from initial biopsy slides alone may guide clinical decision making with regards to diagnostic workup, treatment and surveillance of patients with high risk for PO. Clinicians may use the information gained from self-supervised learning as an adjunct in clinical decision making and assigning pre-test probabilities for patients that might be at higher risk for PO and benefit from further workup and management. (Figure 6e). For example, patients identified at high-risk may be deemed appropriate for pre-operative imaging, more frequent follow up or removal of a primary tumor with complete margin control and enhanced pathologic staging provided by Mohs micrographic surgery(45, 46).

Development of, and access to, large datasets will be crucial to further validate and expand the current study. Ultimately, the ability to assess the risk of PO at time of initial diagnosis could provide the basis to establish and test diagnostic and therapeutic protocols that could ultimately optimize clinical outcome.

## Method

### Dataset

Datasets of shave or punch biopsy specimens were collected from three institutions each with separate IRB approval processes (**Table 1**, Supplementary Figure 1a): 119 slides from 42 patients were collected at New York University (NYU), 95 slides from 95 patients were collected at the University of California San Francisco (UCSF) and 40 slides from 40 patients were collected at the Brigham and Women’s Hospital (BWH). For cases at NYU, multiple slides from a single lesion were analyzed per patient. For slides from BWH and UCSF, single slides of the highest yield resolution were obtained for ease of logistical coordination between sites. Due to lack of information or poor slide quality (e.g., out of focus), fourteen slides were removed from the study (Supplementary Figure 5). To benefit from the best resolution available, whole slide images were scanned on an Aperio AT2 scanner and captured at 0.25 um/pixel at 40 X using JPEG2000 compression and stored as a .svs pyramidal file.

De-identified slides from other collaborating institutions were sent to NYU Langone Health’s Dermatologic Surgery & Cosmetic Associates Office. The scanned images did not contain any patient information. De-identified samples were simply classified as good versus POs. All de-identified physical slides were stored at NYU Langone Health’s Dermatologic Surgery & Cosmetic Associates Office until the analysis was complete. Upon completion of analysis, the slides were returned to the original institution.

Adult patients 18–89 years old with existing slides of biopsy proven cSCC obtained prior to January 1, 2021 were included. Patients were excluded if they were outside the specified age range and had no histological confirmation of cSCC. Patients treated at NYU Langone Health’s Dermatologic Surgery & Cosmetic Associates Office were identified for inclusion by a NYULH study team member based on the above inclusion and exclusion criteria above. Patients at NYU were identified using retrospective chart review of those with poor outcome, and thereon manual review of slides were performed to select those with the best slide quality. Patients treated at collaborating institutions were identified by individuals at those institutions based on the inclusion and exclusion criteria.

Patients were classified as having a PO if the tumor was successfully treated but the tumor came back at any time in the future, either in the form of local recurrence (LR), nodal metastasis (NM), distant metastasis (DM) or if the patient had a disease-specific death (DSD). Otherwise, if no tumor was detected at subsequent visits, the patients were classified as having a good outcome. In total, 119 patients were associated with good outcomes and 44 with POs. For UCSF and NYU, the times to LR, NM, DM or DSD were also available, giving further granularity into the disease-free survival (DFS) analysis. However, this data was not available for the BWH cohort.

### Self-supervised-based analysis

The self-supervised-based study was based on the Histomorphological Phenotype Learning (HPL) through self-supervised learning and community detection pipeline developed by *Quiros et al.* (29) and summarized as follows and in Figure 1 (and Supplementary Figure 5). Using the DeepPATH tools(47), images from the 3 datasets were first tiled (removing those where the background covers more than 75% of the tile, and applying color normalizing using the Reinhard’s method (48), and converted to a h5 file such as each tile fed to the self-supervised pipeline has a field of view of 224 × 224 pixels at a pixel size of about 0.5 um (corresponding to a magnification of 20x, Figure 1a). The 2,069,052 resulting tiles were split such that 40% of the tiles from each dataset were combined and used to train the self-supervised Barlow-Twins algorithm based network(37) (Figure 1b). After training, all the tiles were projected into the *z* tile representation vector of the trained network and are represented by UMAPs(49) and PAGA(50) in this manuscript. As a filtering step, a first Leiden clustering(38) was achieved using a resolution of 7 in order to obtain a large (n=136) number of Histomorphological Phenotype Clusters (HPCs) and over-cluster and increase the chance of having homogeneous HPCs. Those HPCs were visually inspected to identify those containing artifacts (air bubbles, out-of-focus regions, etc. Figure 1c), with the goal to remove from the rest of study the tiles from HPCs representing artifacts. The resulting 1,998,932 tiles were then used for the rest of the study.

After artifact tiles were removed, a final round of Leiden clustering was run using a wide range of Leiden resolutions. Considering the small dataset available, the analyses were done using a 3-fold cross-validation approach to study the variability of the approach while allowing each set to have enough samples. In each fold, a different third was used as a test set, while the remaining tiles are split between training (80%) and validation (20%). To ensure representativity and proper split, folds were generated randomly with a single constraint on the RFS to ensure each train and test sets have similar Kaplan-Meier profiles. After Leiden clustering, each whole slide image (WSI) can be represented by a codebar called a WSI vector representation which describes the distribution of tiles in each HPC. When a patient has more than one slide available, those can also be aggregated into a “patient vector representation”. Logistic and/or Cox proportional hazards regressions have been run using the patient vector representations from the training sets, and evaluated using the validation and test sets left. Similar to the previous study on lung cancer(29), the performance was analyzed on a set of cluster configurations via n-fold cross validation to estimate variability at a given Leiden resolution, the Wald test being used to measure the significance on each regression and using Fischer’s method to combine the p-values. Once done, we locked down a fold for further analysis.

As the resolution parameter r of the Leiden clustering algorithm is increased, the UMAP appears as split into more HPCs (Supplementary Figure 2c, green curve). However, as the number of HPC increases and gets smaller, in terms of average number of tiles, the risk of obtaining institution or patient-specific clusters increases (Supplementary Figure 2c, purple and cyan curves), which would be a sign of over-fitting. Indeed, increasing the number of clusters too much increases the risk of detecting features which are patient or institution specific (which may be caused by the fact that the 3 cohorts were stained in 3 different institutions and scanned on 2 different scanners, or may be related to some other phenotypes specific to natural variations between individuals). However, we are interested in finding common patterns across the three institutions, with enough meaningful (or compact) clusters to describe the diversity of common patterns found in this disease. Therefore, to select the best Leiden cluster resolution for the subsequent analysis of the HPCs, we checked, for each resolution: 1- the average patient and institution presence in HPCs (see details below); 2- the performance of the binary classifier (good versus poor outcome) via the AUC (Area Under the receiver operating Curve) of the logistic regression approach; 3- the performance of the Cox Regression for survival prediction. The average patient presence (Supplementary Figure 2c) is defined as the average percentage of patients present in the HPCs at a given resolution, either counting all patients even if only 1 of their tiles belong to a certain HPC, or using a 1% threshold. Similarly, the institution presence (Supplementary Figure 2c) is defined as the average percentage of institutions present in the HPCs at a given resolution, either counting all institutions, or only counting those with at least 1% of their tiles associated with a given HPC. Despite the small size of our cohort and limited number of institutions involved, it allows us to get a sense of the potential generalization of the study, and these averages will tend to get smaller as the HPCs become more patient or institution specific. We notice that at resolutions higher than r=0.75, these averages decrease, showing that more HPCs become specific and less generalizable across patients and institutions. Binary classification between good and poor outcome was done using all three samples, while survival analysis data was only available for the NYU and UCSF datasets. Those analyses were done using a three-fold cross validation approach (Supplementary Figure 2d–e), using folds consistent with those used for Leiden cluster determination to study report influence on variations between different Leiden clustering runs (Supplementary Figure 5). The logistic and cox regressions were done following the approach detailed in *Quiros et al.* (29). Briefly, WSI vector representations were built for each patient to describe the percentage of tiles associated with each HPC, and center log-ratio transformation was applied to use those in linear models. A three fold cross-validation analysis was performed such as, for each fold, one third of the patients is used as a test set, and the rest is used for the training/validation process. For each fold, the regressions were fit using the training set and assessed with the validation and test sets.

Elastic-net penalty models were used for regression where we optimized the alpha parameter (final value of 0.25) for the logistic regression analysis, and the alpha and l1 ratio parameters (to final values of 0.35 and 0.01 respectively) for the Cox regression analysis. After having locked a cluster configuration, the medium of the hazard predictions on the training set was used to define the threshold between the low and high risk groups used on the test set and shown in Figure 2g. The statistical significance between the two groups is measured using the log rank test and a p-value threshold of 0.05.

### Cluster analysis

UMAPs(49) (Uniform Manifold Approximation and Projection) and PAGA(50) (partition-based graph abstraction) were used to visualize the tile vector representations and resulting Leiden clusters. PAGA provides an additional layer of interpretability by preserving the topology where edges between the nodes denote statistically relevant connectivity between HPCs.

For each HPC, 100 tiles were randomly selected and visually interpreted (blinded from the positions of the HPCs on the PAGA) by three board certified Mohs surgeons and a senior dermatology resident (M.C., S.R.J, R.W.) who freely annotated and labeled each of them (no features to choose from were supplied). Consensus annotations, which arrived if all reviewers agreed with the annotation features, are shown in Supplementary Table 6. Annotations were then mapped into the PAGA (Figure 3), where interesting connections between nodes can be seen despite the pathologists’ annotations having been done without knowledge of the PAGA.

Analyses of the correlation between the clusters and external annotations were done following the HPL pipeline(29): SHAP (SHapley Additive exPlanations) and Forest plots were used to evaluate how each HPC affects the log odds ratio of patients. The SHAP values were calculated across each test set of 3-fold cross-validation analyses. The Forest plots are based on the log hazard ratio of Cox proportional hazards model over the train sets of a 3-fold cross-validation. The coefficients were averaged across fold and combined p-values with Fisher’s combined probability test. Correlations with pathologic diagnostic and type of recurrence (LR or overall metastases) was achieved using Spearman’s rank correlation with a significance threshold of 0.01 on the p-values (adjusted with the Benjamini/Hochberg(51) method for false discovery rate). Overall metastases include both nodal and distant ones.

### Supervised Analysis

To explore whether the performance of the outcome prediction in a supervised manner, we used DeepPATH(47) and followed an approach comparable to the one used to predict response to Melanoma treatment in *Johannet et al.* (22), training inception v3(52) twice at a magnification of 20x: first to automatically segment the slides, second to predict the outcome from selected segmented regions. For the segmentation, a 3 and 5-class network were trained. In the 3-class approach explored, the network was trained to identify the following classes: regions of interest, artifacts and other features (muscle, bone, cartilage, hair follicles, nerve…). The goal was therefore to simply be able to sort out the artifacts and other features regions judged irrelevant by the team to later predict outcome. In the 5-class approach, the network was designed to split more precisely the “regions of interest”, and it was therefore trained to identify the following classes: invasive SCC, *in-situ* SCC, normal epidermis, artifacts and other features. Next, we trained a network to study the predictability of the good versus poor outcome using the regions of interest only, or using the invasive SCC only.

## Supplementary Material

Supplement 1

## Data Availability

Reasonable requests for cohort data may be addressed to the corresponding authors.

## References

[1] Lomas A, Leonardi-Bee J, Bath-Hextall F. A systematic review of worldwide incidence of nonmelanoma skin cancer. Br. J. Dermatol. 2012;166(5):1069–1080. 22251204 10.1111/j.1365-2133.2012.10830.x

[2] Waldman A, Schmults C. Cutaneous Squamous Cell Carcinoma. Hematol. Oncol. Clin. North Am. 2019;33(1):1–12. 30497667 10.1016/j.hoc.2018.08.001

[3] Stang A, Incidence and mortality for cutaneous squamous cell carcinoma: comparison across three continents. J. Eur. Acad. Dermatol. Venereol. 2019;33 Suppl 8(Suppl 8):6–10. 10.1111/jdv.15967 PMC6913879 31833607

[4] Leiter U, Incidence, Mortality, and Trends of Nonmelanoma Skin Cancer in Germany. Journal of Investigative Dermatology 2017;137(9):1860–1867. 28487088 10.1016/j.jid.2017.04.020

[5] van Lee CB, Recurrence rates of cutaneous squamous cell carcinoma of the head and neck after Mohs micrographic surgery vs. standard excision: a retrospective cohort study. Br. J. Dermatol. 2019;181(2):338–343. 30199574 10.1111/bjd.17188

[6] Lansbury L, Interventions for non-metastatic squamous cell carcinoma of the skin: systematic review and pooled analysis of observational studies. BMJ 2013;347(v04 1):f6153–f6153. 24191270 10.1136/bmj.f6153 PMC3816607

[7] Rogers HW, Incidence Estimate of Nonmelanoma Skin Cancer (Keratinocyte Carcinomas) in the U.S. Population, 2012. JAMA Dermatol. 2015;151(10):1081–1086. 25928283 10.1001/jamadermatol.2015.1187

[8] Karia PS, Han J, Schmults CD. Cutaneous squamous cell carcinoma: Estimated incidence of disease, nodal metastasis, and deaths from disease in the United States, 2012. Journal of the American Academy of Dermatology 2013;68(6):957–966. 23375456 10.1016/j.jaad.2012.11.037

[9] Eigentler TK, Survival of Patients with Cutaneous Squamous Cell Carcinoma: Results of a Prospective Cohort Study. Journal of Investigative Dermatology 2017;137(11):2309–2315. 28736229 10.1016/j.jid.2017.06.025

[10] Stevenson ML, Use of Adjuvant Radiotherapy in the Treatment of High-risk Cutaneous Squamous Cell Carcinoma With Perineural Invasion. JAMA Dermatol. 2020;156(8):918–921. 32609298 10.1001/jamadermatol.2020.1984 PMC7330824

[11] Lewis KG, Weinstock MA. Trends in nonmelanoma skin cancer mortality rates in the United States, 1969 through 2000. J. Invest. Dermatol. 2007;127(10):2323–2327. 17522705 10.1038/sj.jid.5700897

[12] American Cancer Society. Facts & Figures 2023. Paperpile https://paperpile.com/app/p/47ca1717-2267-0081-9d88-750bb97346e9. cited April 6, 2023

[13] Karia PS, Comparison of Tumor Classifications for Cutaneous Squamous Cell Carcinoma of the Head and Neck in the 7th vs 8th Edition of the AJCC Cancer Staging Manual. JAMA Dermatol. 2018;154(2):175–181. 29261835 10.1001/jamadermatol.2017.3960 PMC5815880

[14] Amin MB, The Eighth Edition AJCC Cancer Staging Manual: Continuing to build a bridge from a population-based to a more “personalized” approach to cancer staging. CA: A Cancer Journal for Clinicians 2017;67(2):93–99. 28094848 10.3322/caac.21388

[15] Gupta N, Identifying Brigham and Women’s Hospital stage T2a cutaneous squamous cell carcinomas at risk of poor outcomes. J. Am. Acad. Dermatol. 2022;86(6):1301–1308. 34864111 10.1016/j.jaad.2021.11.046

[16] Group Work, Guidelines of care for the management of cutaneous squamous cell carcinoma. J. Am. Acad. Dermatol. 2018;78(3):560–578. 29331386 10.1016/j.jaad.2017.10.007 PMC6652228

[17] Jennings L, Schmults CD. Management of high-risk cutaneous squamous cell carcinoma. J. Clin. Aesthet. Dermatol. 2010;3(4):39–48. PMC2921745 20725546

[18] Wysong A, Validation of a 40-gene expression profile test to predict metastatic risk in localized high-risk cutaneous squamous cell carcinoma. J. Am. Acad. Dermatol. 2021;84(2):361–369. 32344066 10.1016/j.jaad.2020.04.088

[19] Zhao G, AXIN2 and SNAIL expression predict the risk of recurrence in cutaneous squamous cell carcinoma after Mohs micrographic surgery. Oncology Letters 2020; doi:10.3892/ol.2020.11324 PMC7039156 32194711

[20] Yanofsky VR, Mercer SE, Phelps RG. Histopathological variants of cutaneous squamous cell carcinoma: a review. J. Skin Cancer 2011;2011:210813. 21234325 10.1155/2011/210813 PMC3018652

[21] Yacob F, Weakly supervised detection and classification of basal cell carcinoma using graph-transformers on whole slide images 2023; doi:10.21203/rs.3.rs-2499377/v1 PMC10169852 37160953

[22] Johannet P, Using Machine Learning Algorithms to Predict Immunotherapy Response in Patients with Advanced Melanoma. Clin. Cancer Res. 2021;27(1):131–140. 33208341 10.1158/1078-0432.CCR-20-2415 PMC7785656

[23] Kim RH, Deep Learning and Pathomics Analyses Reveal Cell Nuclei as Important Features for Mutation Prediction of BRAF-Mutated Melanomas. J. Invest. Dermatol. 2022;142(6):1650–1658.e6. 34757067 10.1016/j.jid.2021.09.034 PMC9054943

[24] Comes MC, A deep learning model based on whole slide images to predict disease-free survival in cutaneous melanoma patients. Sci. Rep. 2022;12(1):20366. 36437296 10.1038/s41598-022-24315-1 PMC9701687

[25] Knuutila JS, Identification of metastatic primary cutaneous squamous cell carcinoma utilizing artificial intelligence analysis of whole slide images. Sci. Rep. 2022;12(1):9876. 35701439 10.1038/s41598-022-13696-y PMC9197840

[26] Sali R, Deep Learning for Whole-Slide Tissue Histopathology Classification: A Comparative Study in the Identification of Dysplastic and Non-Dysplastic Barrett’s Esophagus. Journal of Personalized Medicine 2020;10(4):141. 32977465 10.3390/jpm10040141 PMC7711456

[27] Dimitriou N, Arandjelović O, Caie PD. Corrigendum: Deep Learning for Whole Slide Image Analysis: An Overview. Front. Med. 2020;7:419. 10.3389/fmed.2020.00419 PMC7466414 32974358

[28] Shmatko A, Artificial intelligence in histopathology: enhancing cancer research and clinical oncology. Nat Cancer 2022;3(9):1026–1038. 36138135 10.1038/s43018-022-00436-4

[29] Quiros AC, Self-supervised learning in non-small cell lung cancer discovers novel morphological clusters linked to patient outcome and molecular phenotypes. arXiv [cs.CV] 2022;http://arxiv.org/abs/2205.01931. cited

[30] Chen Z, Optimization of deep learning models for the prediction of gene mutations using unsupervised clustering. Hip Int. 2023;9(1):3–17. 10.1002/cjp2.302 PMC9732687 36376239

[31] Kim J, Author Correction: Unsupervised discovery of tissue architecture in multiplexed imaging. Nat. Methods 2022;19(12):1662. 36414761 10.1038/s41592-022-01717-7

[32] Chen RJ, Scaling Vision Transformers to Gigapixel Images via Hierarchical Self-Supervised Learning. 2022 IEEE/CVF Conference on Computer Vision and Pattern Recognition (CVPR) 2022; doi:10.1109/cvpr52688.2022.01567

[33] Poon AIF, Sung JJY. Opening the black box of AI-Medicine. J. Gastroenterol. Hepatol. 2021;36(3):581–584. 33709609 10.1111/jgh.15384

[34] Laak J van der, Deep learning in histopathology: the path to the clinic. Nature Medicine 2021;27(5):775–784. 10.1038/s41591-021-01343-4 33990804

[35] Guidotti R, A Survey of Methods for Explaining Black Box Models. ACM Comput. Surv. 2018;51(5):1–42.

[36] Rajpurkar P, AI in health and medicine. Nature Medicine 2022;28(1):31–38. 10.1038/s41591-021-01614-0 35058619

[37] Zbontar J, Barlow Twins: Self-Supervised Learning via Redundancy Reduction. In: Meila M, Zhang T eds. Proceedings of the 38th International Conference on Machine Learning. PMLR; 2021:12310–12320

[38] Traag VA, Waltman L, van Eck NJ. From Louvain to Leiden: guaranteeing well-connected communities. Sci. Rep. 2019;9(1):5233. 30914743 10.1038/s41598-019-41695-z PMC6435756

[39] Zakhem GA, Association of Patient Risk Factors, Tumor Characteristics, and Treatment Modality With Poor Outcomes in Primary Cutaneous Squamous Cell Carcinoma. JAMA Dermatology 2023;159(2):160. 36576732 10.1001/jamadermatol.2022.5508 PMC9857763

[40] Brancaccio G, Risk Factors and Diagnosis of Advanced Cutaneous Squamous Cell Carcinoma. Dermatol Pract Concept 2021;11(Suppl 2):e2021166S. 34877074 10.5826/dpc.11S2a166S PMC8609959

[41] Que SKT, Zwald FO, Schmults CD. Cutaneous squamous cell carcinoma: Incidence, risk factors, diagnosis, and staging. J. Am. Acad. Dermatol. 2018;78(2):237–247. 29332704 10.1016/j.jaad.2017.08.059

[42] Rowe DE, Carroll RJ, Day CL Jr. Prognostic factors for local recurrence, metastasis, and survival rates in squamous cell carcinoma of the skin, ear, and lip. Implications for treatment modality selection. J. Am. Acad. Dermatol. 1992;26(6):976–990. 1607418 10.1016/0190-9622(92)70144-5

[43] Campoli M, Brodland DG, Zitelli J. A prospective evaluation of the clinical, histologic, and therapeutic variables associated with incidental perineural invasion in cutaneous squamous cell carcinoma. J. Am. Acad. Dermatol. 2014;70(4):630–636. 24433872 10.1016/j.jaad.2013.11.034

[44] Schmults CD, Factors Predictive of Recurrence and Death From Cutaneous Squamous Cell Carcinoma. JAMA Dermatology 2013;149(5):541. 23677079 10.1001/jamadermatol.2013.2139

[45] Gibson FT, Perioperative imaging for high-stage cutaneous squamous cell carcinoma helps guide management in nearly a third of cases: A single-institution retrospective cohort. J. Am. Acad. Dermatol. [published online ahead of print: January 18, 2023]; doi:10.1016/j.jaad.2023.01.012 36681240

[46] Canavan TN, A cohort study to determine factors associated with upstaging cutaneous squamous cell carcinoma during Mohs surgery. J. Am. Acad. Dermatol. 2023;88(1):191–194. 35378171 10.1016/j.jaad.2022.03.055

[47] Coudray N, Classification and mutation prediction from non-small cell lung cancer histopathology images using deep learning. Nat. Med. 2018;24(10):1559–1567. 30224757 10.1038/s41591-018-0177-5 PMC9847512

[48] Reinhard E, Color transfer between images. IEEE Comput. Graph. Appl. July-Aug 2001;21(5):34–41.

[49] McInnes L, UMAP: Uniform Manifold Approximation and Projection. Journal of Open Source Software 2018;3(29):861.

[50] Wolf FA, PAGA: graph abstraction reconciles clustering with trajectory inference through a topology preserving map of single cells. Genome Biology 2019;20(1). doi:10.1186/s13059-019-1663-x PMC6425583 30890159

[51] Benjamini Y, Hochberg Y. Controlling the False Discovery Rate: A Practical and Powerful Approach to Multiple Testing. Journal of the Royal Statistical Society: Series B (Methodological) 1995;57(1):289–300.

[52] Szegedy C, Rethinking the Inception Architecture for Computer Vision. 2016 IEEE Conference on Computer Vision and Pattern Recognition (CVPR) 2016; doi:10.1109/cvpr.2016.308

