# Supplementary material for: Self-supervised artificial intelligence predicts recurrence, metastasis and disease specific death from primary cutaneous squamous cell carcinoma at diagnosis
Source: Res Sq. 2023 Dec 13:rs.3.rs-3607399. Preprint. [Version 1] doi: 10.21203/rs.3.rs-3607399/v1 (PMC10760225; doi:10.21203/rs.3.rs-3607399/v1)
Supplement: Supplement 1 [file NIHPPrs3607399v1-supplement-1.pdf]

Supplementary Figure 1. Available datasets and overall workflow of the study. a. Proportion of patients with good and poor outcome from the different institutions (New York University: NYU; University of California, San Francisco: UCSF; and Brigham and Women's Hospital: BWH) and Kaplan-Meier curves of disease-free survival (DFS) events for patients from NYU and UCSF. **b.** The three datasets were studied using 2 different deep-learning approaches: a self-supervised approach (Histological Phenotype Learning pipeline, HPL), and a supervised pipeline (DeepPATH).

Supplementary Figure 2. Analysis of the tile vector representation z and its clustering with the Leiden algorithm. a. Training (red) and validation (blue) loss after training the Barlow-Twins model (60 epochs, error corresponds to standard deviation on a 3 fold-cross validation test, and curves shown after exponential moving average done on 1 epoch period (about 1,400 iterations)) **b.** UMAP of the tile vector representation from the three different institutions is shown in a separate plot, with each dot representing a tile, and each color representing a different patient. **c.** Effect of the Leiden resolution on the number of clusters (HPC) obtained (green circles, right-hand side axis), measure of the average presence of institutions in each HPC (if at least 1 tile of an institution is present, purple triangles, left-hand side axis - and if at least 1% of that institution's tiles are present, purple diamonds, left-hand side axis), and measure of the average presence of patients in each HPC (if at least 1 tile of a patient is present, cyan triangles, left-hand side axis - and if at least 1% of a patient's tiles are present, cyan diamonds, left-hand side axis). **d.** Impact of Leiden resolution on the prediction of the outcome as a binary label (good vs poor) using Log-regression. **e.** Impact of Leiden resolution on the prediction of the outcome as a continuous label (disease-free survival, DFS) using Cox-regression (For panels B, C and D, error bars show the standard deviation on a three-fold cross-validation runs).

Supplementary Figure 3. Prediction of the outcome as a binary label using self-supervised vs supervised approach. a. Performance of the self-supervised approach using a three-fold cross-validation approach and log regression on the patient's HPC content **b.** Associated Forest plot showing the top clusters associated with those predictions and the associated p-values. **c.**

Performance of the slide segmentation supervised classifier based on 3 or 5 classes. **d.** Supervised binary outcome predictions run on either sets of regions of interest (invasive tumor + *in-situ* + normal regions) or on invasive cutaneous squamous cell carcinoma regions only.

Supplementary Figure 4. Performance of supervised classifier vs self-supervised. a. Comparison of the ROC curves from experiments shown in **Supplementary Figure 3** for binary classification of patients with poor vs good outcome. **b.** Kaplan-Meier curves for survival on all stages, on stage T2 (AJCC staging) and T2a (BWH staging) extrapolated from probabilities assigned to each patient by the 4-fold cross-validation supervised classifier I (trained on invasive, normal and SCCIS regions). **c.** Kaplan-Meier curves for survival on all stages, on stage T2 (AJCC staging) and T2a (BWH staging) extrapolated from probabilities assigned to each patient by the 4-fold cross-validation supervised classifier II (trained exclusively on invasive tumor regions).

Supplementary Figure 5. Slide selection and self-supervised processing workflow.

Supplementary Figure 6. Examples of randomly selected tiles for each HPC. All tiles are shown after Reinhard's color normalization (48).

Supplementary Table 1. Clinical outcomes for the available cohorts from academic institutions Brigham and Women's Hospital (BWH), New York University (NYU) and University of California San Francisco (UCSF).

	BWH	NYU	UCSF
**Number of slides, n**	40	119	85
**Number of patients, n**	40	42	85
**Type of outcome per patient, n**			
Good outcome	20	31	58
Poor outcome	20	7	27
**Type of poor outcome at follow-up, n**			
Local recurrence	0	1	23
Metastasis	20	6	5
Nodal metastasis ONLY	NA	NA	3
Nodal & Distant metastasis	NA	NA	1
Metastasis & Local recurrence	0	0	1

NA = not available. BWH did not include information on the specific type of metastasis. NYU did not differentiate the type of metastasis.

Supplementary Table 2. AUC and PR for the supervised segmentation of H&E slides (with CIs being confidence intervals)

Task	Class	AUC [CIs]	Baseline for AUC	Precision/Recall [CIs]	Baseline for PR
3-classes	Regions of	0.973 [0.965-	0.5	0.997 [0.995-	0.928

segmentation	interest	0.981]		0.998]	
	Artifacts	0.990 [0984-0.994]	0.5	0.906 [0.883-0.928]	0.052
	other features	0.926 [0.896-0.954]	0.5	0.641 [0.557-0.721]	0.020
	micro	0.996 [0995-0.997]	0.5	0.996 [0.995-0.997]	0.333
	macro	0.964 [0.951-0.976]	0.5	NA	NA
4-classes segmentation	Invasive SCC	0.942 [0.942-0.950]	0.5	0.993 [0.991-0.994]	0.899
	SCCIS	0.902 [0.875-0.902]	0.5	0.140 [0.075-0.226]	0.010
	Normal epidermis	0.987 [0.985-0.993]	0.5	0.645 [0.552-0.733]	0.019
	Artifacts	0.982 [0.983-0.985]	0.5	0.859 [0.827-0.890]	0.052
	other features	0.898 [0.888-0.932]	0.5	0.556 [0.467-0.639]	0.020
	micro	0.991 [0.991-0.992]	0.5	0.990 [0.989-0.992]	0.200
	macro	0.943 [0.937-0.949]	0.5	NA	NA

Supplementary Table 3. Metrics for the supervised segmentation of H&E slides

Task	Specificity	Accuracy	Precision	recall/sensitivity	F1-score
3-class segmentation	0.996	0.950	0.970	0.491	0.652
5-class segmentation	0.975	0.903	0.944	0.515	0.667

Supplementary Table 4. AUC and PR for the supervised and self-supervised prediction of outcome as a binary label (good vs poor outcome); Standard Error Means on 3 and 4 fold classifications are shown

Task	AUC [SEM]	Precision / Recall for good outcome [SEM]	Baseline for Precision / Recall for good outcome	Precision / Recall for poor outcome [SEM]	Baseline for Precision / Recall for poor outcome
Supervised classifier on regions of interest	0.637 [0.047]	0.777 [0.035]	0.67	0.498 [0.015]	0.33
Supervised classifier on invasive SCC	0.671 [0.009]	0.802 [0.004]	0.67	0.576 [0.016]	0.33
Self-supervised classifier	0.689 [0.043]	0.827 [0.032]	0.67	0.515 [0.026]	0.33

Supplementary Table 5. Metrics for the supervised and self-supervised prediction of outcome as a binary label, after applying a classification threshold using the Youden method

Task	Specificity	Accuracy	Precision	Recall / Sensitivity	F1-score
Supervised classifier on regions of interest	0.482	0.563	0.448	0.721	0.529
Supervised classifier on invasive SCC	0.565	0.615	0.457	0.725	0.553

Self-supervised classifier	0.737	0.635	0.841	0.582	0.669

Supplementary Table 6. Visual annotations of HPCs

HPC ID	Description
0	Well differentiated keratinocytes
1	Poorly differentiated keratinocytes
2	Poorly differentiated keratinocytes, mitoses
3	Nonspecific hyperkeratosis
4	Well differentiated keratinocytes
5	Pleomorphic and poorly differentiated keratinocytes with mitoses
6	Deep invasion of pleomorphic and poorly differentiated keratinocytes with mitoses
7	Well differentiated keratinocytes, mitoses
8	Nonspecific hyperkeratosis
9	Cartilage
10	Pleomorphic, poorly differentiated keratinocytes
11	Pleomorphic, poorly differentiated keratinocytes with mitoses and dense inflammatory infiltrate
12	Well differentiated keratinocytes
13	Pleomorphic keratinocytes, presence of subcutaneous fat and glandular tissue
14	Well differentiated keratinocytes, inflammatory infiltrate
15	Well differentiated keratinocytes

16	Hyperkeratosis, well differentiated keratinocytes
17	Well differentiated keratinocytes
18	Poorly differentiated with deep invasion
19	Pleomorphic keratinocytes with mitoses and presence of dense inflammatory infiltrate
20	Pleomorphic, poorly differentiated keratinocytes with mitoses
21	Poorly differentiated, pleomorphic keratinocytes, cartilage
22	Dense inflammation
23	Sebocytes
24	Hyperkeratosis, well differentiated keratinocytes with mitoses
25	Poorly differentiated keratinocytes with deep invasion

Supplementary Figure 1

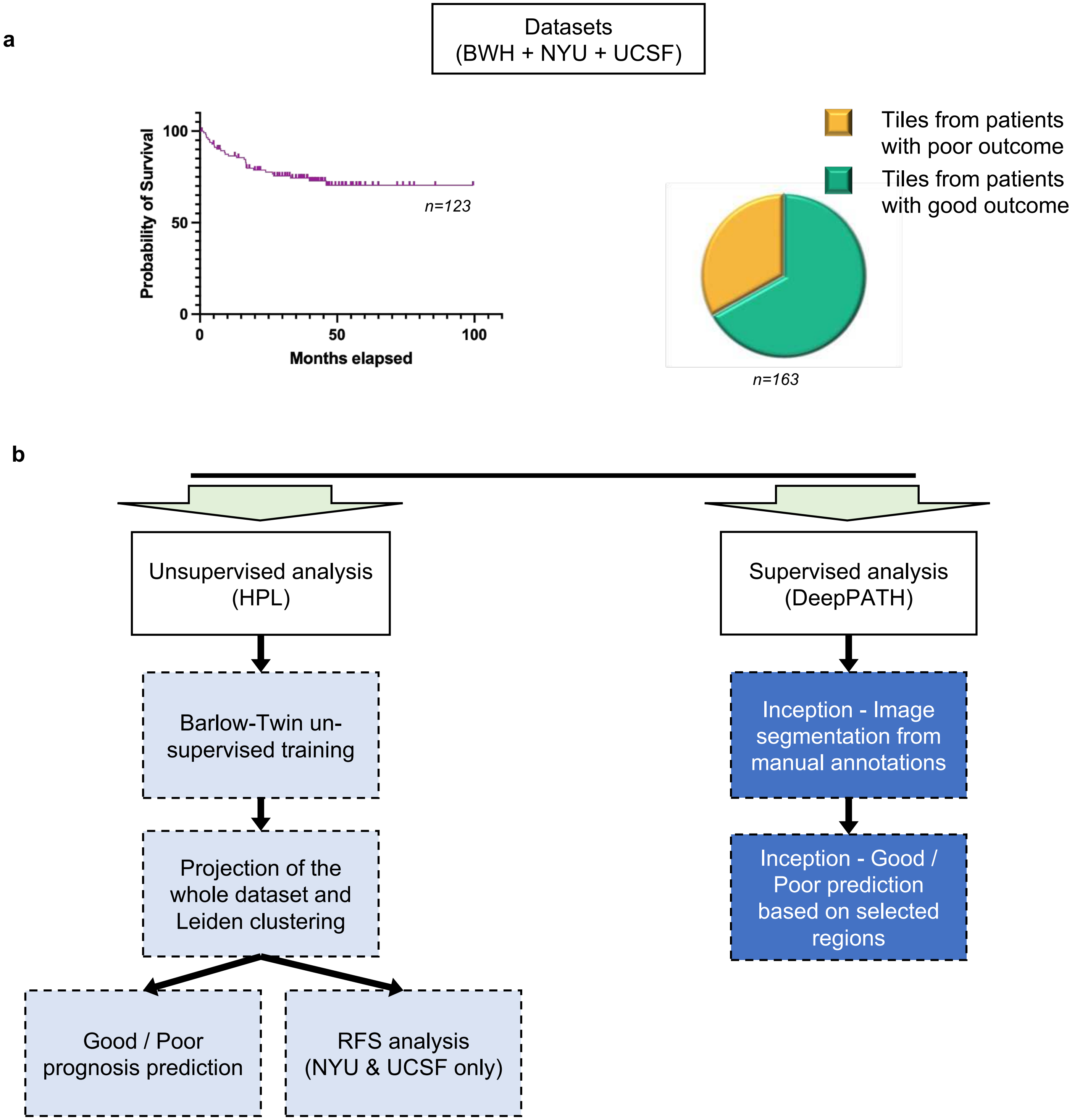

Supplementary Figure 1. Available datasets and overall workflow of the study. a. Proportion of patients with good and poor outcome from the different institutions (New York University: NYU; University of California, San Francisco: UCSF; and Brigham and Women's Hospital: BWH) and Kaplan-Meier curves of disease-free survival (DFS) events for patients from NYU and UCSF. **b.** The three datasets were studied using 2 different deep-learning approaches: an unsupervised approach (Histological Phenotype Learning pipeline, HPL), and a supervised pipeline (DeepPATH).

Supplementary Figure 2

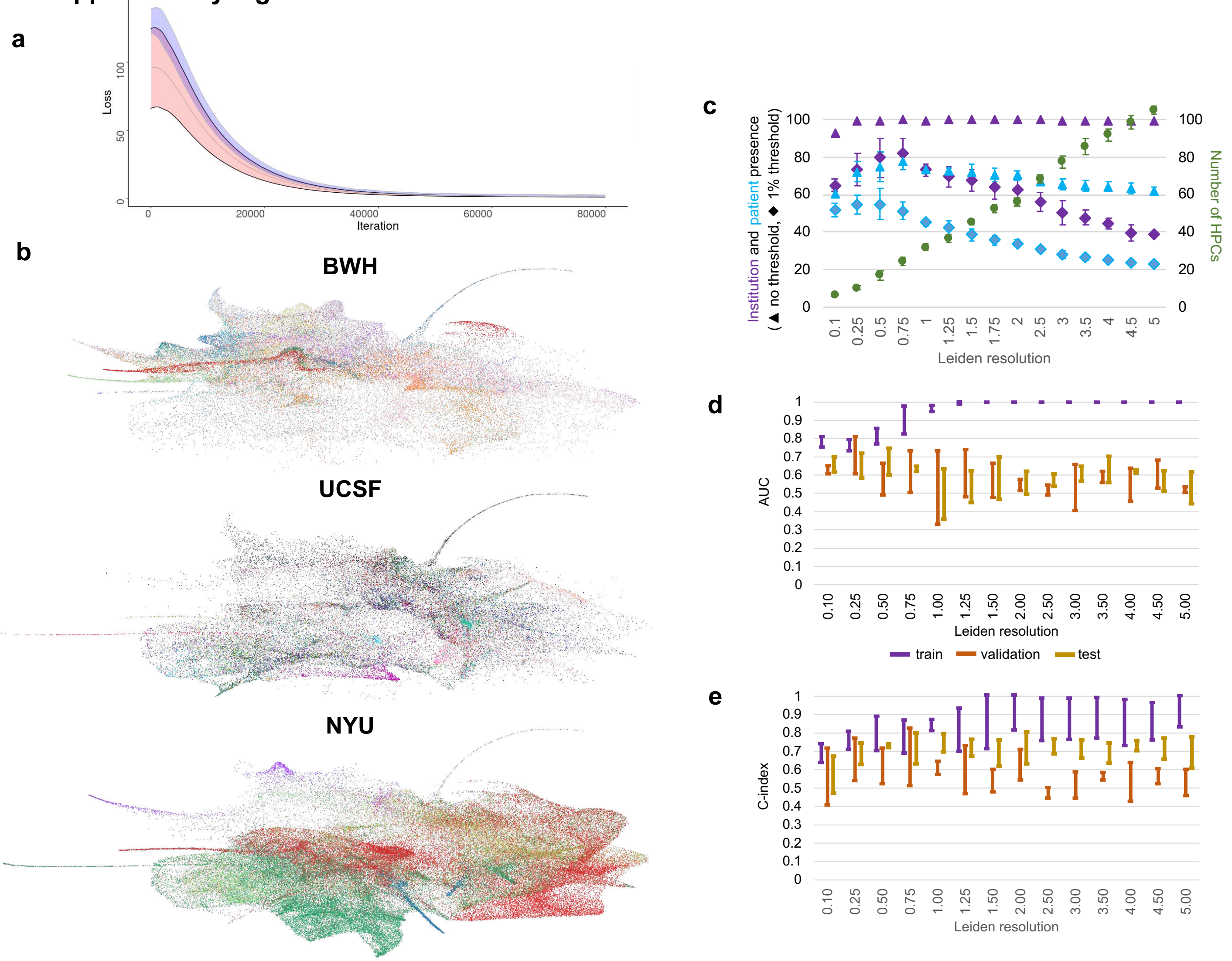

Supplementary Figure 2. Analysis of the tile vector representation z and its clustering with the Leiden algorithm. a. Training (red) and validation (blue) loss after training the Barlow-Twins model (60 epochs, error corresponds to standard deviation on a 3 fold-cross validation test, and curves shown after exponential moving average done on 1 epoch period (about 1,400 iterations)) **b.** UMAP of the tile vector representation from the three different institutions is shown in a separate plot, with each dot representing a tile, and each color representing a different patient. **c.** Effect of the Leiden resolution on the number of clusters (HPC) obtained (green circles, right-hand side axis), measure of the average presence of institutions in each HPC (if at least 1 tile of an institution is present, purple triangles, left-hand side axis - and if at least 1% of that institution's tiles are present, purple diamonds, left-hand side axis), and measure of the average presence of patients in each HPC (if at least 1 tile of a patient is present, cyan triangles, left-hand side axis - and if at least 1% of a patient's tiles are present, cyan diamonds, left-hand side axis). **d.** Impact of Leiden resolution on the prediction of the outcome as a binary label (good vs poor) using Log-regression. **e.** Impact of Leiden resolution on the prediction of the outcome as a continuous label (disease-free survival, DFS) using Cox-regression (For panels B, C and D, error bars show the standard deviation on a three-fold cross-validation runs).

Supplementary Figure 3

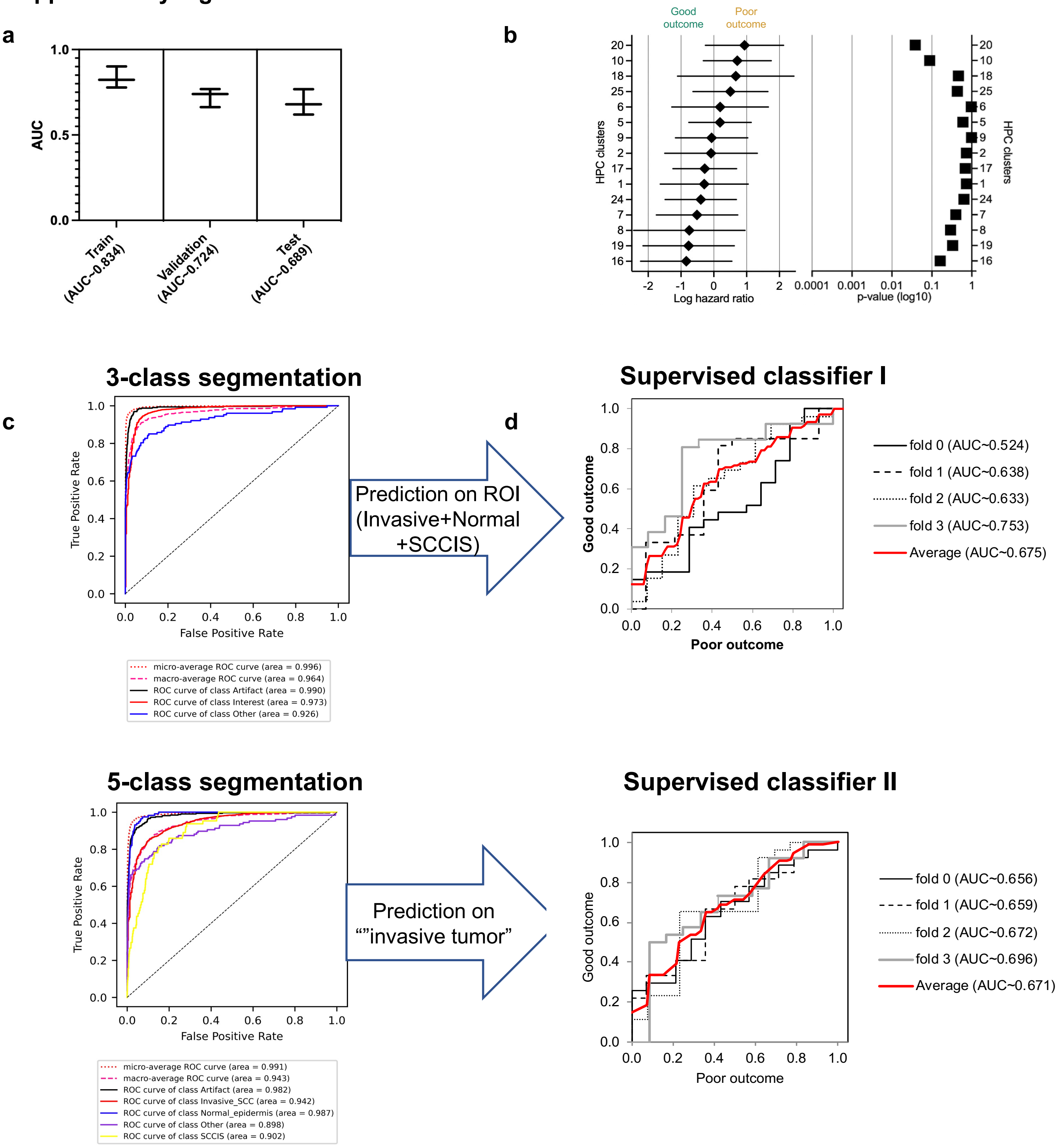

Supplementary Figure 3. Prediction of the outcome as a binary label using unsupervised vs supervised approach. a. Performance of the unsupervised approach using a three-fold cross-validation approach and log regression on the patient’s HPC content **b.** Associated Forest plot showing the top clusters associated with those predictions and the associated p-values. **c.** Performance of the slide segmentation supervised classifier based on 3 or 5 classes. **d.** Supervised binary outcome predictions run on either sets of regions of interest (invasive tumor + *in-situ* + normal regions) or on invasive cutaneous squamous cell carcinoma regions only.

Supplementary Figure 4

a Supervised classifiers I and II (binary) vs self-supervised (binary log regression) ROC

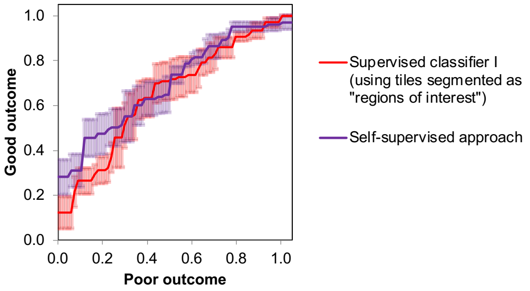

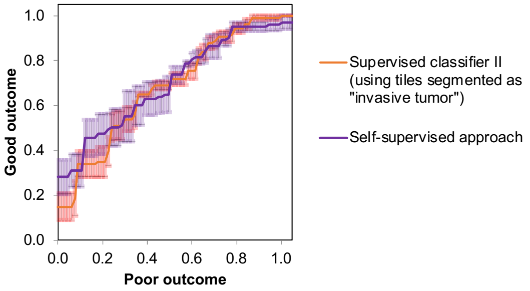

b Supervised classifier I (binary) vs self-supervised (Cox regression) ROC

Survival on all stages

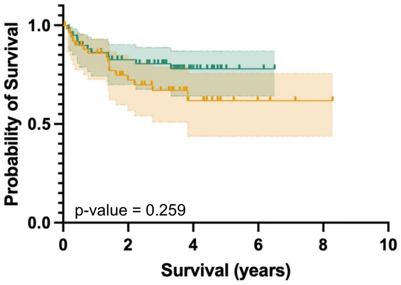

Survival on stage T2 (AJCC staging system)

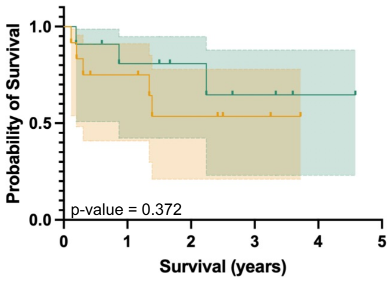

Survival on stage T2a (BWH staging system)

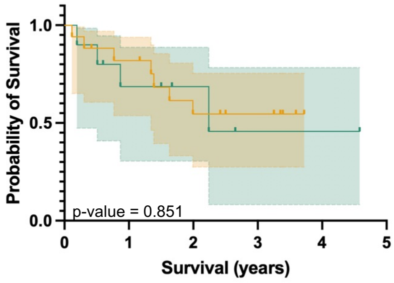

c Supervised classifier II (binary) vs self-supervised (Cox regression) ROC

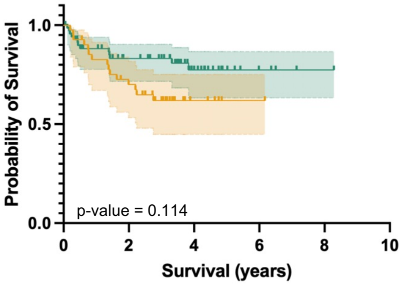

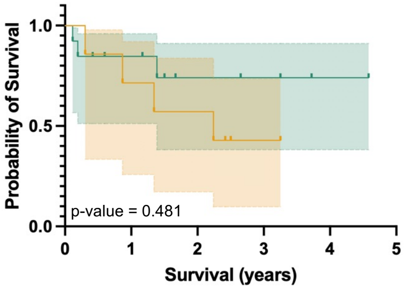

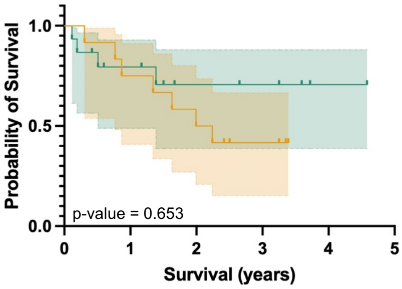

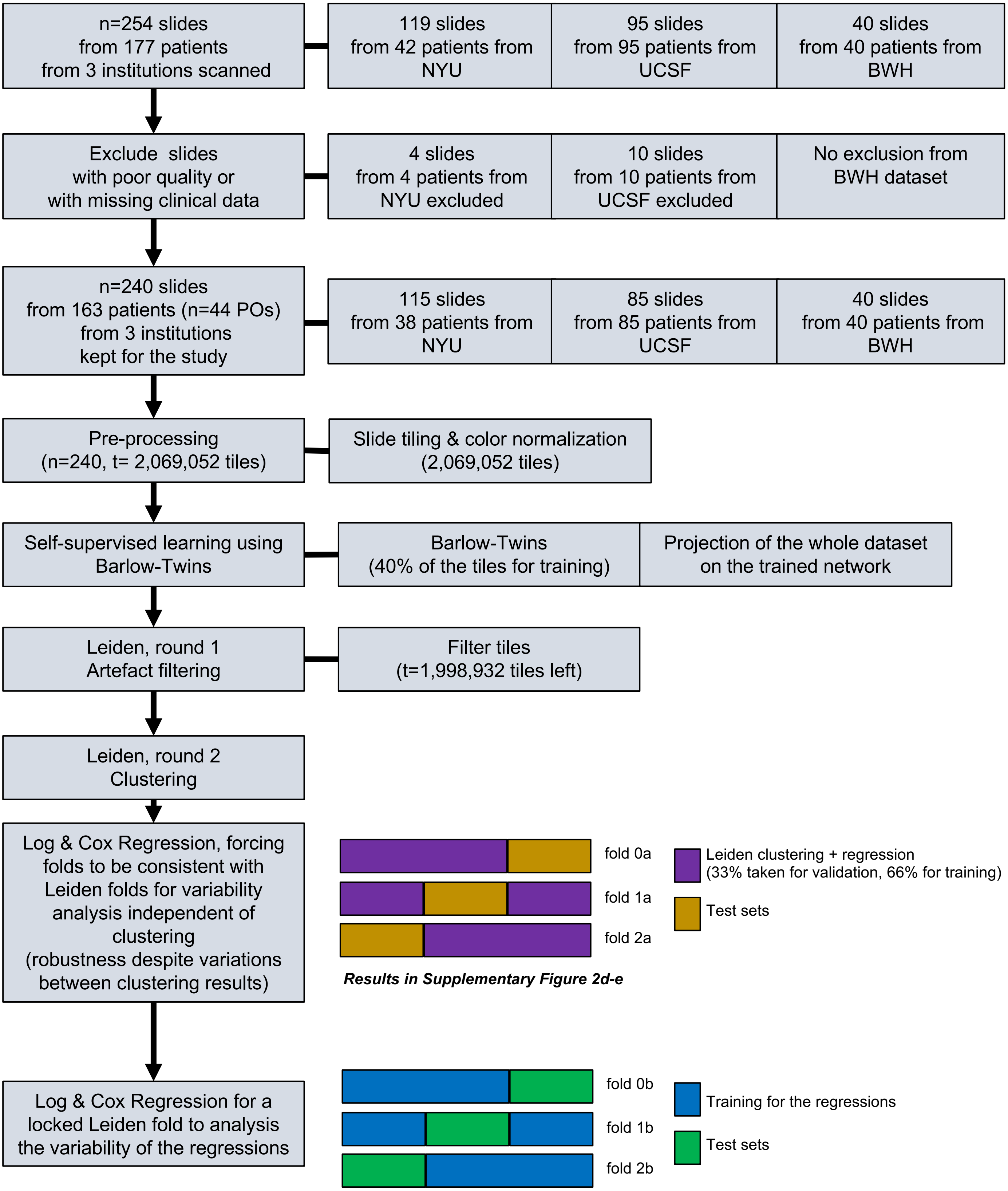

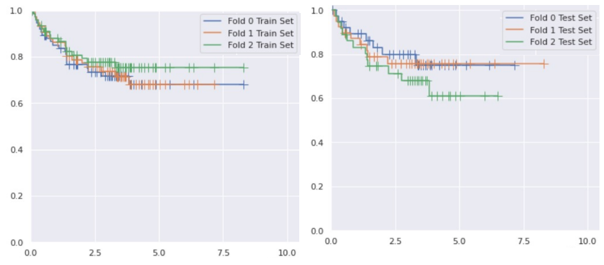

Supplementary Figure 5. Slide selection and self-supervised processing workflow.

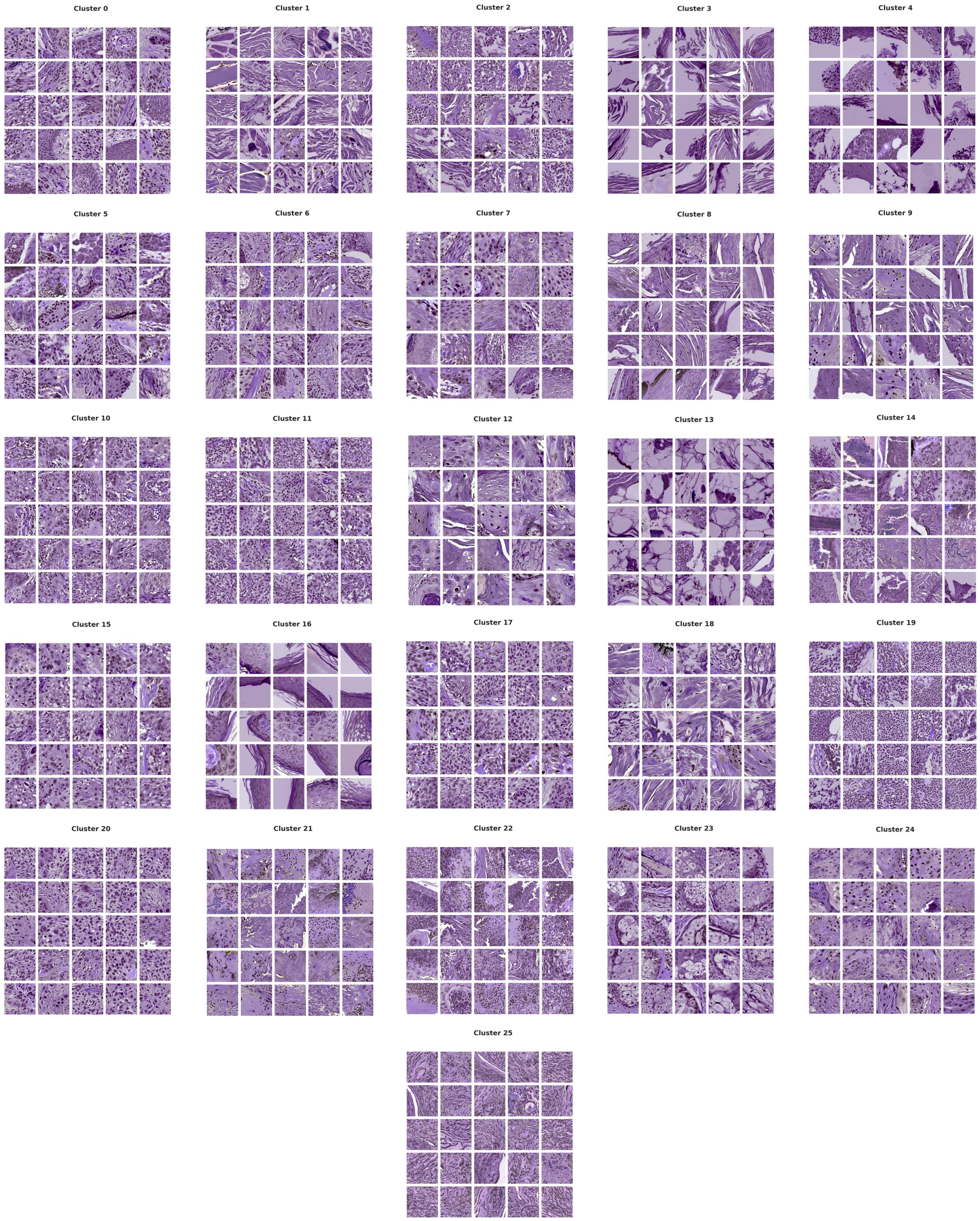

Supplementary Figure 6. Examples of randomly selected tiles for each HPC. All tiles are shown after Reinhard's color normalization (48).
